# RNA interference in cytochrome P450 monooxygenase (CYP) gene results in reduced insecticide resistance in *Megalurothrips usitatus* Bagnall

**DOI:** 10.3389/fphys.2023.1130389

**Published:** 2023-03-27

**Authors:** Weiyi Chen, Zhaoyang Li, Chenyan Zhou, Asad Ali, Shaukat Ali, Jianhui Wu

**Affiliations:** ^1^ Key Laboratory of Bio-Pesticide Innovation and Application, Engineering Research Center of Biological Control, College of Plant Protection, South China Agricultural University, Guangzhou, China; ^2^ Department of Agriculture, Abdul Wali Khan University, Mardan, Pakistan

**Keywords:** *Megalurothrips usitatus* (Bagnall), insecticide resistance, transcriptome, cytochrome P-450, RNA interference

## Abstract

Genes of the cytochrome P450 (CYP450) superfamily are known to be involved in the evolution of insecticide resistance. In this study, the transcriptomes of two *Megalurothrips usitatus* Bagnall (Thysanoptera: Thripidae) strains (resistant and susceptible) were screened for detoxification genes. *MusiDN2722* encodes a protein composed of 504 amino acid residues with a relative molecular mass of 57.3 kDa. Multiple sequence alignment and phylogenetic analysis showed that *MusiDN2722* is a member of the CYP450 family and has characteristics of the conserved CYP6 domain shared by typical CYP450 family members. RT-qPCR (real-time quantitative polymerase chain reaction) analysis showed that *MusiDN2722* was upregulated in the acetamiprid-resistant strain compared with the susceptible strain (*p* < 0.05), and the relative expression level was significantly higher at 48 h after exposure than at 24 h after exposure. The interference efficiency of the injection method was higher than that of the membrane-feeding method. Silencing of *MusiDN2722* through RNA interference significantly increased the sensitivity of *M. usitatus* to acetamiprid. Overall, this study revealed that *MusiDN2722* plays a crucial role in the resistance of *M. usitatus* to acetamiprid. The findings will not only advance our understanding of the role of P450s in insecticide resistance but also provide a potential target for the sustainable control of destructive pests such as thrips.

## 1 Introduction

The bean flower thrip *Megalurothrips usitatus* Bagnall (Thysanoptera: Thripidae) is a major pest of leguminous crops grown in southern China, especially in Hainan Province (Huang et al., 2018; Yang et al., 2021). A range of synthetic insecticides are extensively used for *M. usitatus* management, but its high reproduction rate and short generation time have induced the development of insecticide resistance. Acetamiprid, a second-generation nicotinoid insecticide, acts through antagonization of nAChR receptors, thus hindering nerve impulse transmission across the central nervous system of insects (Elbert et al., 2008; Phogat et al., 2022). In recent years, various thrip species (*Frankliniella occidentalis*, *Thrips tabaci*, *Thrips hawaiiensis*, *Scolothrips takahashii*, and *M. usitatus*) have developed resistance to acetamiprid in different regions of the world (Mori and Gotoh, 2001; Chen and Yuan, 2011; Fu et al., 2016; Nazemi et al., 2016; Wang et al., 2016; Han et al., 2017; Zuo et al., 2017; Fu et al., 2019; Lin et al., 2021). The mechanism of resistance to neonicotinoids is mainly attributed to two factors: target insensitivity and increased metabolic detoxification (Puinean et al., 2010; Ihara et al., 2020). Enhanced detoxification mediated by insect cytochrome P450 monooxygenases (CYPs) is a major mechanism of resistance development.

CYP450s play an essential physiological role in the growth, development, and reproduction of insects (Scott, 1999; Cui et al., 2016). They are also involved in the biosynthesis and degradation pathways of endogenous compounds (such as pheromones, 20-hydroxyecdysone, and juvenile hormone (JH)) (Cifuentes et al., 2012; Roberto et al., 2017; Xu et al., 2020). Cytochrome P450 (CYP450) is the main detoxification enzyme in insects, and its action is considered to be one of the main mechanisms underlying resistance of insects to insecticides (Berge et al., 1998). CYP450 might be involved in resistance and cross-resistance mechanisms in the MEAM1 whitefly (*Bemisia tabaci* Gennadius) (Zhou et al., 2020). Three P450 genes (*CYP6CY14*, *CYP6DC1*, and *CYP6CZ1*) have been found to be involved in the development of resistance to acetamide in *Aphis gossypii* (Farman et al., 2020). By knocking out *CYP4PR1*, which is highly expressed in epidermal tissues, the susceptibility of pyrethroid-resistant *Triatoma infestans* can be increased (Dulbecco et al., 2021). Enhanced detoxification mediated by CYPs is the main mechanism of insecticide resistance development in *F. occidentalis* and *Thrips palmi* (Espinosa et al., 2005; Bao et al., 2014). However, the involvement of P450 genes in the development of insecticide resistance in an insect strain with an extremely high degree of resistance to neonicotinoids has not been elucidated in detail.

In this study, a highly acetamiprid-resistant *M. usitatus* strain (established in the laboratory through consecutive selection for 40 generations) was subjected to transcriptome analysis, followed by cloning of the P450 gene *MusiDN2722* to study the role of this gene in the development of acetamiprid resistance in *M. usitatus.* These results will provide basic information on the mechanism of neonicotinoid resistance in *M. usitatus* and thus can help with the formulation of management strategies for acetamiprid-resistant populations of *M. usitatus* in the field.

## 2 Materials and methods

### 2.1 Insect-rearing

Two *M. usitatus* Bagnall strains (one acetamiprid-resistant (AcR) and one susceptible (SS)) were used in this study. The SS strain was collected from Nanbin Farm, Sanya City, Hainan Province, in 2008 and was reared for 60 generations in the laboratory without exposure to any insecticide. The AcR strain was established from the SS population through continuous exposure to acetamiprid for 40 generations, using the leaf dip bioassay method (Rueda and Shelton, 2003). Both strains were reared on fresh cowpea pods in the laboratory at 26°C ± 1°C, under a photoperiod of 14:10 h (light:dark).

### 2.2 Bioassays

The median lethal concentration was determined using the leaf tube film method. Acetamiprid (25% water dispersible granules) was used in a commercially available formulation. Five graded concentrations of acetamiprid were used. Cowpea pods were dipped for 15 s in the designated concentration of insecticide or distilled water (the latter as a control) and placed in the shade until air dried. Emerging adult insects were transferred to the beans. Bioassays were performed in the laboratory at 26°C ± 1°C, under a photoperiod of 12:12 h (light:dark). Each concentration was performed in triplicate, and mortality was assessed after 2 days. LC_50_ (lethal concentration 50%) values were calculated via probit analysis using the SPSS software package (LeOra Software Inc., Berkeley, CA, United States). Resistance factor (RF) was estimated at LC_50_ as RR = LC_50_ of the AcR strain/LC_50_ of the SS strain; the 95% CI for RR was calculated following Preisler and Robertson (1989).

### 2.3 Transcriptome analysis and P450 gene selection

A total of 500 adult females of both the susceptible and resistant strains were collected for three biological replicates. Sample processing, extraction, and metabolite detection for transcriptome analysis were performed by Suzhou PANOMIX Biomedical Tech Co., Ltd. (Suzhou, China), following standard procedures, and the fragments per kilobase of transcript per million mapped reads (FPKM) of the assembled transcripts was calculated. Transcript expression abundance was calculated using the FPKM method (Mortazavi et al., 2008). Benjamini–Hochberg correction of the *p*-value for multiple tests was applied using the false discovery rate (FDR). FDR ≤0.001 and absolute value of the log2 ratio ≥2 were the thresholds for determining significance of differences in gene expression (Itai et al., 2001). Given the large number of P450s in the *M. usitatus* transcriptome, we first characterized gene expression using real-time quantitative polymerase chain reaction (RT-qPCR). Our goal was to identify genes with consistent differential expression.

### 2.4 Construction and identification of a recombinant plasmid with *MusiDN2722*

The SteadyPure Agarose Gel DNA Purification Kit (Accurate Biotechnology, China) was used to purify and recycle the cloned *MusiDN2722* PCR products. The primers used are listed in Supplementary Table S1. The following procedure was adopted to combine the vector components in a 5-μL reaction system. First, the reaction mixture was blended gently and placed in a PCR instrument at 25°C for 5 min. A measure of 5 ml of the transformation product was centrifuged for 1 min, and the entire bacterial solution was subsequently used to cover the Luria–Bertani solid culture. Next, 100 µl of the PCR product was mixed with Trans1-T1 phage-resistant chemically competent cells and aseptically coated on a Luria–Bertani (LB)/ampicillin plate. After growth, preparation, and plasmid DNA analysis, the plate was incubated at 37°C for 12 h; subsequently, a colony was harvested. The colony was cultured in 5 mL of LB liquid medium with ampicillin; the cells were cultured overnight at 37°C. Thereafter, white monoclonals were collected in 10 μL of sterile water and vortexed. A measure of 1 µl of the mixture was mixed with 20 μL of PCR mixture, and positive clones were identified with M13 forward and M13 reverse primers. After these steps, the recombinant plasmid from the positive bacterial fluid was sequenced by Shanghai Sangon Biological Company to determine the clone. The GenBank nucleotide sequence was subtyped and homologously analyzed.

### 2.5 RNA extraction and RT-qPCR

Total RNA was extracted from 300 adults and nymphs of the AcR and SS strains of *M. usitatus* using the Total RNA TRIzol Extractor (Sangon Biotech, China, Shanghai). cDNA was constructed from the total RNA using the PrimeScript™ RT Reagent Kit with gDNA Eraser (TaKaRa, Tokyo, Japan). RT-qPCR was performed in CFX96 TOUCH (Bio-Rad) using TB Green^®^ Premix Ex Taq™ II (Tli RNaseH Plus; TaKaRa, Japan). Gene-specific primers were designed using Premier 5.0 and synthesized by Sangon Biotech Co., Ltd. (Shanghai, China). Experiments were performed thrice with different RNA preparations for each strain. The following cycling conditions were used: 95°C for 30 s; 40 cycles of 95°C for 5 s, 60°C for 30 s, 60°C for 30 s; and 95°C for 1 s for plate reading. Reaction fluorescence was continuously monitored after the cycling protocol using the dissociation temperature of the PCR products at a temperature transition rate of 0.1°C/s to generate a melting curve. Relative gene expression was calculated using the 2^−ΔΔCT^ method (Pfaffl, 2001). The RT-qPCR product was resolved via 1.0% agarose gel electrophoresis, and a DNA fragment of approximately 500 bp was obtained. These results indicated that P450 was expressed in both SS and AcR strains of *M. usitatus*.

### 2.6 Bioinformatic analysis

The sequencing results were submitted to the NCBI (National Center for Biotechnology Information), and the target gene sequences were predicted using the open reading frame (ORF) and conserved domain. The amino acid sequence of the protein encoded by *MusiDN2722* was predicted and analyzed using bioinformatics software applications. The physicochemical properties of the target protein were predicted using ProtParam software (SIB, Swiss Institute of Bioinformatics). The transmembrane region of the target protein was predicted using the TMHMM-2.0 online tool (Department of Health Technology). The signal peptide of the target protein was predicted using the Signal P5.0 server. Finally, the phylogenetic relationships of the target proteins were predicted using MEGA 7.

### 2.7 RNA interference

Specific primers for dsRNA synthesis were designed based on the cDNA sequence of *MusiDN2722* and the fragment sequence of a green fluorescent protein (GFP) containing T7 polymerase promoter sequences at both ends. Approximately 50 newly emerged 3-day-old adult females were collected and placed in a specially designed device. Ten biological replicates were set up. Two approaches were employed for RNA interference: membrane feeding and microinjection of dsRNA. In the membrane-feeding approach, the mouth of a tube was covered with a thin film of BuddyTape (Aglis, Japan). Thereafter, 30 μL of dsRNA was added to the membrane and sealed with Parafilm (Sangon Biotech). After 2 consecutive days of feeding, live adults were collected and half of them were subjected to fluorescence qPCR to verify the silencing efficiency. The remaining half of the live adults were treated with acetamiprid at LC_50_ for bioassays. In the microinjection approach, dsRNA was injected using a microinjector between the mesothoracic shield plate and abdominal segment shield plate into female adult worms.

### 2.8 Data analysis

All results are expressed in the form mean ± standard error; IBM SPSS Statistics 20 software (SPSS, Chicago, IL, United States) was used for statistical analyses. A Student's *t*-test or one-way analysis of variance (ANOVA) was used to compare the differences between samples or among multiple samples in RT-qPCR and bioassays, respectively. Differences were considered significant at *p* > 0.05.

## 3 Results

### 3.1 Analysis of expression profile

After 40 generations of selection, the screening acetamiprid concentration was determined to be 4,700 mg/L, and the LC_50_ values of acetamiprid for the SS and AcR strains were 85.676 and 1439.425 mg/L, respectively. The AcR strain developed a moderate level of resistance to acetamiprid, with a 16.78-fold resistance ratio.

**FIGURE 1:**
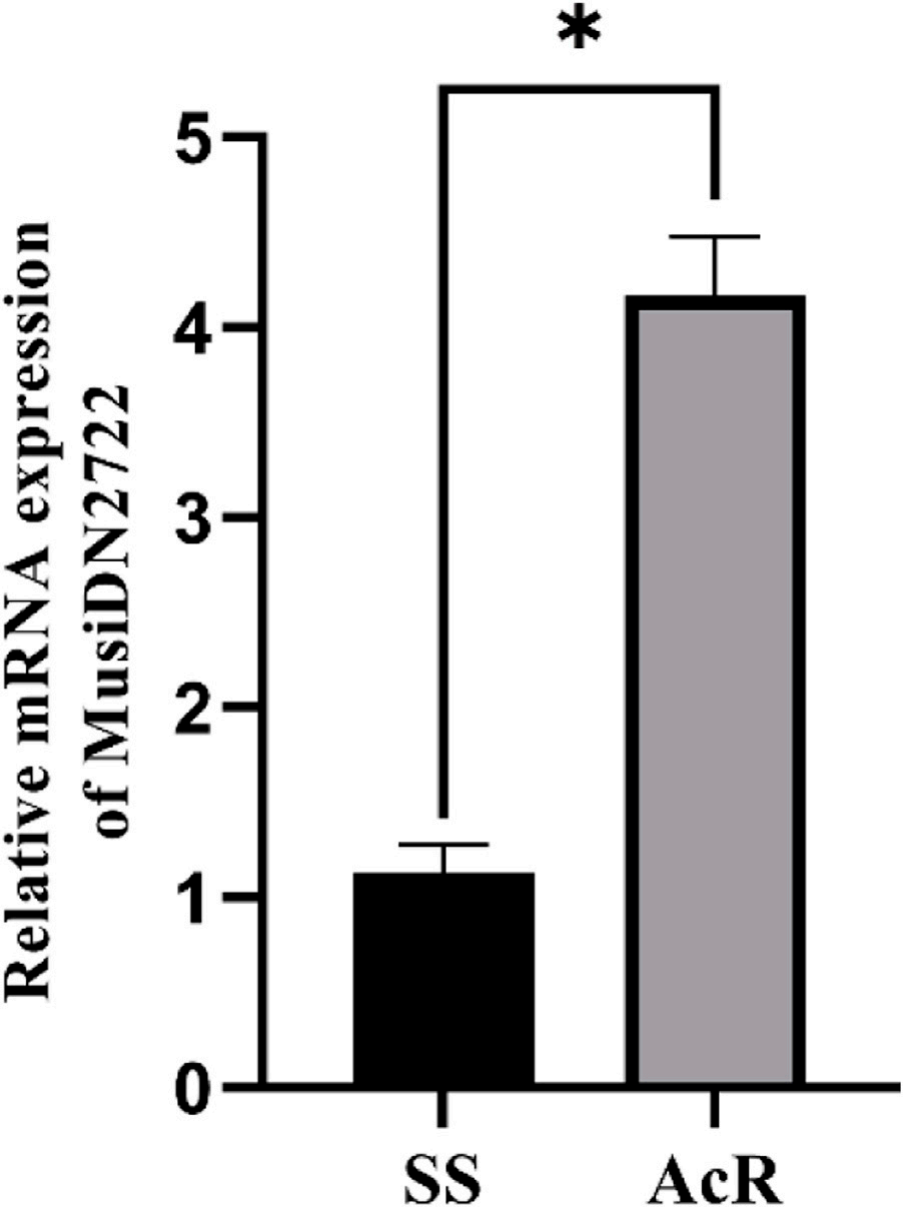
Expression of *MusiDN2722* in two strains. *significant difference (*p* < 0.05).

**FIGURE 2:**
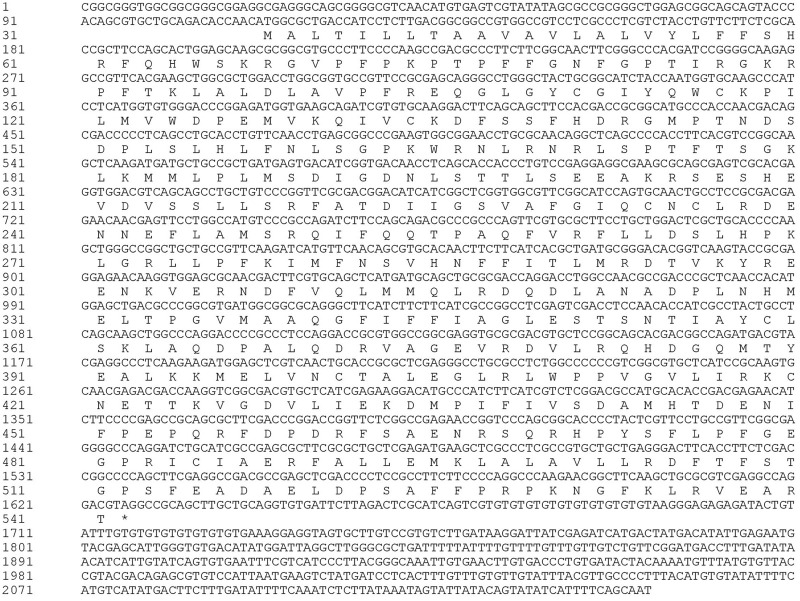
Nucleotide and deduced amino acid sequence of *MusiDN2722*.

The RT-qPCR analysis showed that the expression of *MusiDN2722* was 4.17 times higher in the AcR strain than in the SS strain (Figures 1, 2). Transcriptomic sequencing analysis of the susceptible and resistant strains (SS and AcR) showed that there were more than 40 million original sequences. The Trinity software tool was used to concatenate the filtered sequences, and 21,740 unigenes were obtained (Supplementary Figure S1); the total length was 38343948 bp. The results of differential expression analysis based on the FPKM values showed that there were 167 upregulated and 139 downregulated genes in AcR strains compared with those in the SS strain (Supplementary Figure S2). The results of GO (Gene Ontology) term enrichment analysis indicated enrichment of genes under the following terms: binding, catalytic activity, cellular process, metabolic process, single-organism process, cell, cell junction, and organelle (Supplementary Figure S3). The results of KEGG (Kyoto Encyclopedia of Genes and Genomes) pathway enrichment analysis indicated enrichment of genes under the following terms: carbohydrate metabolism, translation, folding, sorting and degradation, signal transduction, transport and catabolism, and the endocrine system (Supplementary Figure S4). The P450 superfamily genes were identified in the transcriptome analysis of the two strains of *M. usitatus*. We list the top 10 CYP450 genes with a fold difference greater than 2 in Tables 1 and 2. We selected a gene of interest (TRINITY_DN2722_c0_g1) for full characterization in this study; this gene was named *MusiDN2722*.

**TABLE 1:** Genes annotated as cytochrome P450.

Gene ID	Log_2_Fold	*p*-value	NR-annotation
TRINITY_DN4541_c3_g1	17.42010275	1.98E-01	Cytochrome p450
TRINITY_DN899_c0_g1	5.393972239	5.31E-01	Cytochrome P450 6k1
TRINITY_DN2722_c0_g1	3.54462	1.46E-01	Cytochrome p450
TRINITY_DN12852_c0_g1	3.452228214	6.82E-01	Cytochrome P450 6a2-like isoform X1
TRINITY_DN9313_c0_g1	2.904800048	9.00E-01	Cytochrome P450
TRINITY_DN1932_c1_g2	2.827139838	2.88E-01	Cytochrome P450 4C1
TRINITY_DN222_c4_g1	2.409297348	5.25E-01	Cytochrome P450
TRINITY_DN27581_c0_g2	2.230435956	7.39E-01	Cytochrome P450 9e2-like
TRINITY_DN22321_c0_g1	2.200350449	3.25E-01	Cytochrome P450 301a1

**TABLE 2:** Median lethal concentration (LC_50_) of acetamiprid against *M. usitatus*.

Strain	LC_50_(mg/L)	95%FL	Chi-square	Slope (±SE)	Resistance ratio
SS	85.676	60.57–111.61	0.602	1.045 ± 0.142	1.0
AcR	1439.425	1064.52–3316.60	1.637	0.663 ± 0.135	16.78

### 3.2 Bioinformatics analysis

#### 3.2.1 Prediction of physicochemical properties

**FIGURE 3:**
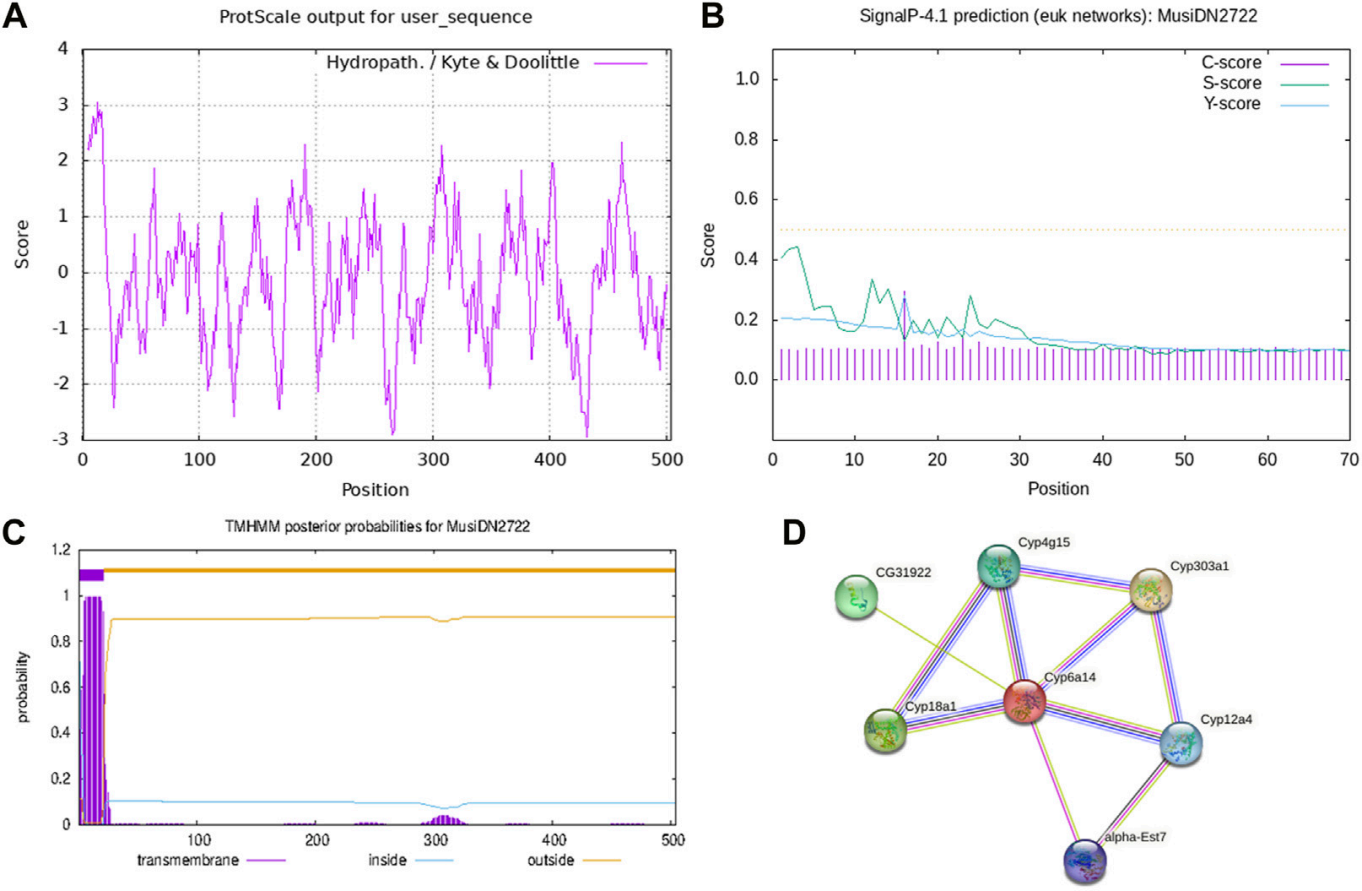
Domain analysis of *MusiDN2722*. **(A)** Hydrophobicity analysis. **(B)** Signal peptide analysis. **(C)** Prediction of transmembrane domain structures. **(D)** Prediction of protein.

Analysis using the ProtParam tool showed that the molecular formula of *MusiDN2722* was C_2583_H_4033_N_699_O_723_S_28_. The full-length cDNA sequence of *MusiDN2722* was 2075 bp, with the CYP6 conserved domain of the P450 superfamily. Sequence analysis showed that the ORF of *MusiDN2722* was 1515 bp and this encodes a protein of 504 amino acids with a relative molecular weight of 57.3 KDa and a PI (isoelectric point) of 11.06. The total number of negatively charged residues (Asp + Glu) carried by the protein was 61. The total number of positively charged residues (Arg + Lys) was 109. The total mean value of hydrophilicity was −0.128, and the instability coefficient was 39.59, indicating that *MusiDN2722* is hydrophilic and stable (Figure 3A). Analysis using the SignalP 4.1 and TMHMN 2.0 tools revealed that *MusiDN2722* had no signal peptide (Figure 3B) but had one transmembrane structure, which was in the range of 2–21 bp (Figure 3C).

#### 3.2.2 *MusiDN2722* target protein prediction

We analyzed *MusiDN2722* using the STRING protein interaction database; the results showed that *MusiDN2722* may be related to the cytochrome C oxide subunit proteins Cyp303a (NP_0012859777.1), CYP18a1 (CAL69954.1), CYP4g15 (NP_727531.2), CYP12a4 (NP_650783.2), CG31922 (NP_722687.1), and alpha-EST7 (NP_524261.1) (Figure 3D).

#### 3.2.3 Multiple sequence alignment and phylogenetic tree analysis of *MusiDN2722*

**FIGURE 4:**
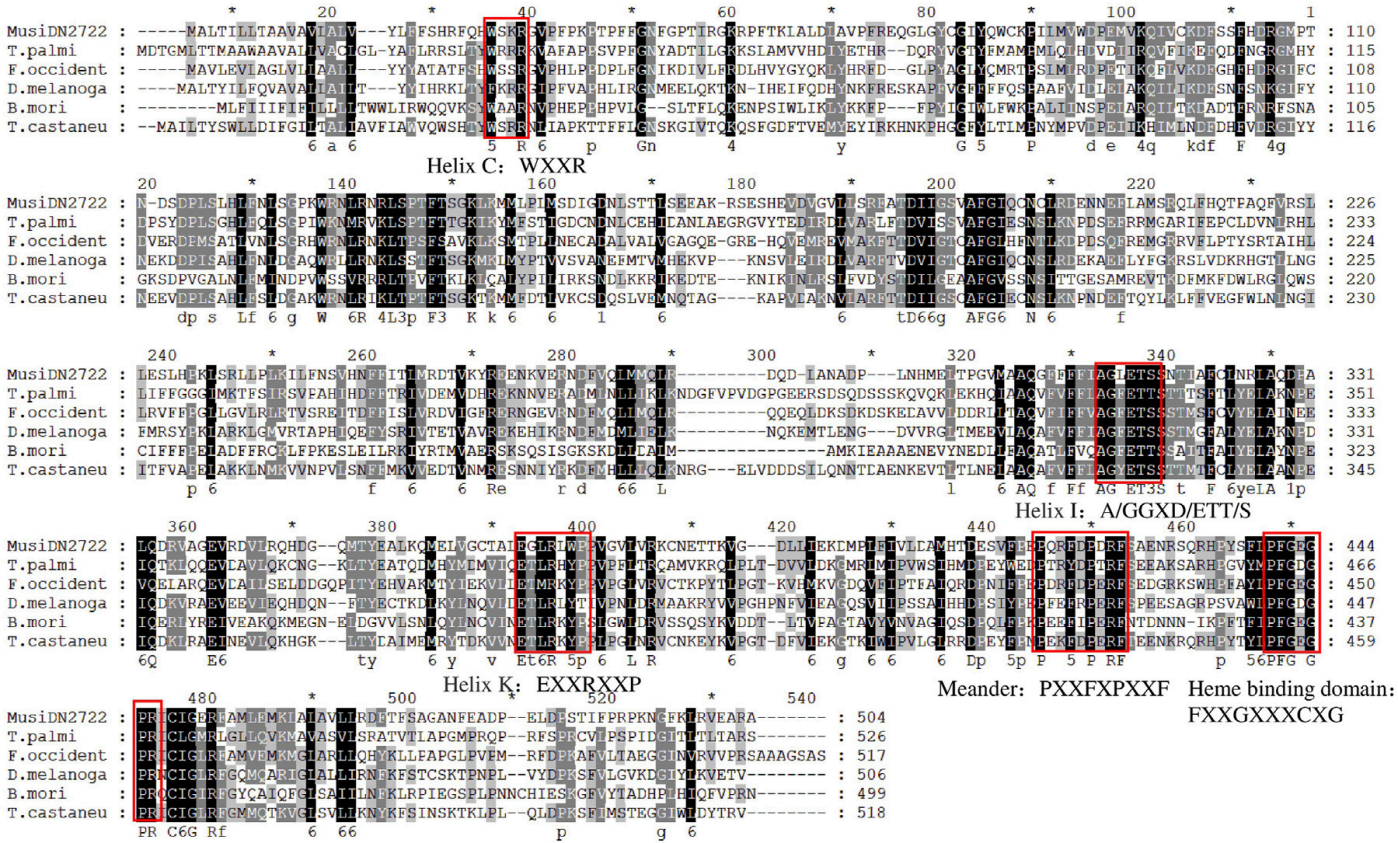
Multiple sequence alignment of *M. usitatus* cytochrome P450s. Included species and corresponding Genbank accession numbers: *T. palmi*, *Thrips palmi* (XP_034255739.1 CYP6a2); *F. occident*, *Frankliniella occidentalis* (KAE8752265.1 CYP6); *D. melanoga*, *Drosophila melanogaster* (AAF58185.2 CYP6a8); *B. mori*, *Bombyx mori* (XP_037874445.1 CYP6k1).

Multi-sequence comparison results showed that six CYP450s had a common CYP450 feature sequence located in the spiral C region (“WXXR” sequence), the spiral K region (“EXXRXXP” sequence), and the Meander region (“PPXXF” sequence). Above the CYP450s, the characteristic sequence “FXXGXXXCXG” appeared in the heme-binding region and the sequence “A/GGXD/ETT/S” in the spiral I region. Phylogenetic analysis revealed that *MusiDN2722* was most closely related to CYP450 from *F. occidentalis* (Figure 4). The amino acid sequences of 37 CYP450 proteins from 26 other insects were analyzed phylogenetically (Figure 4). Based on the results of the evolutionary tree, the *MusiDN2722* gene cloned in this study belongs to the CYP6 family. The evolutionary distance between *MusiDN2722* and *FoccCYP6A14* was small; they shared 84.8% amino acid similarity and were clustered in the same branch as *Thrips palmi TpCYP6A13* and *TpCYP6A2*. This finding suggests that *MusiDN2722* is evolutionarily conserved.

### 3.3 *MusiDN2722* expression at different developmental stages and in different tissues of *M. usitatus*

**FIGURE 5:**
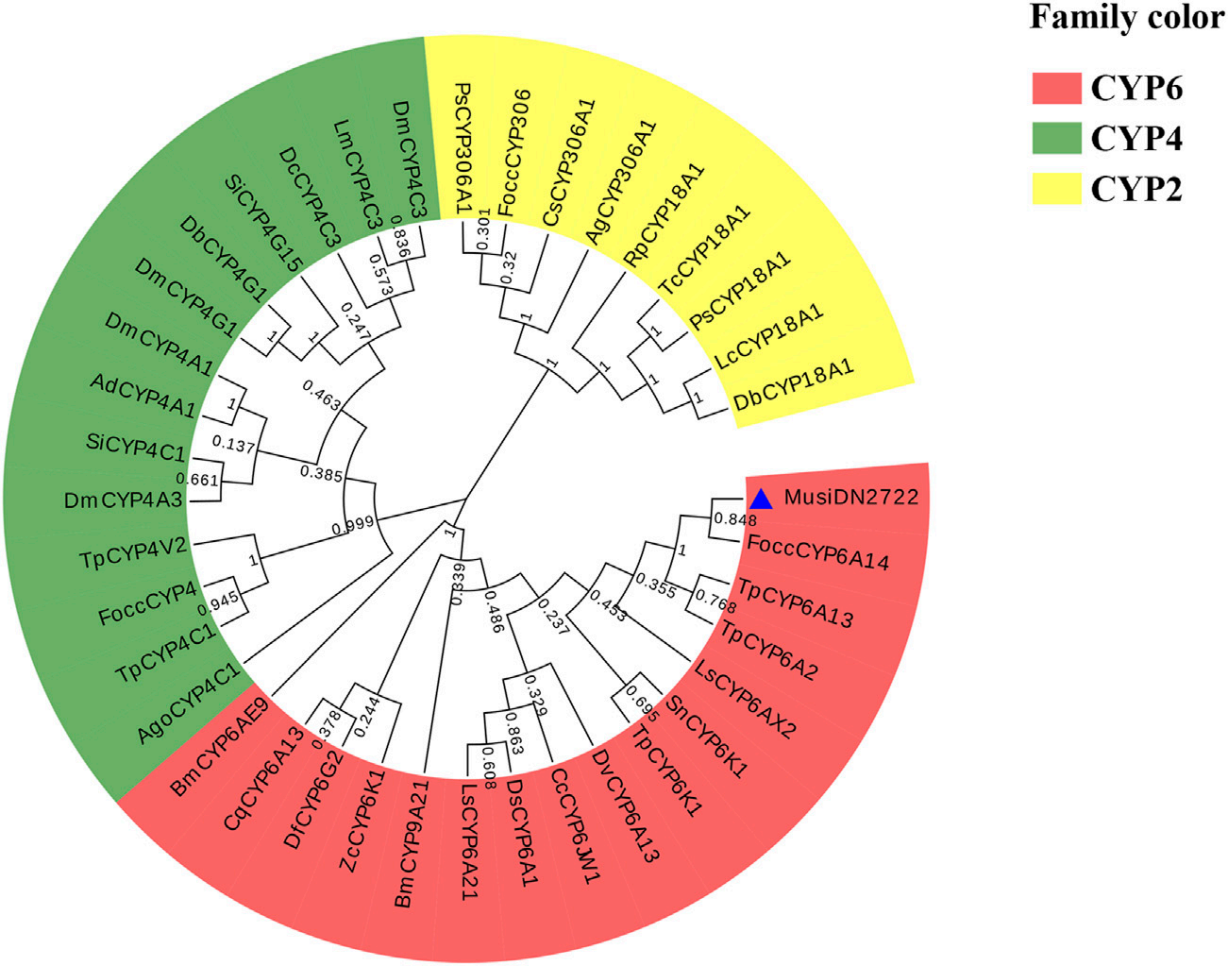
Phylogenetic analysis of *MusiDN2722*. Focc, *Frankliniella occidentalis*; Tp, *Thrips palmi*; Sn, *Schistocerca nitens*; Cc, *Ceratitis capitata*; Ls, *Laodelphax striatellus*; Dv, *Diabrotica virgifera*; Bm, *Bombyx mori*; Ds, *Drosophila simulans*; Df, *Drosophila ficusphila*; Zc, *Zerene cesonia*; Cq, *Culex quinquefasciatus*; Ls, *Lucilia sericata*; Bm, *Bombyx mori*; Tc, *Tribolium castaneum*; Lc, *Lucilia cuprina*; Rp, *Rhopalosiphum padi*; Cs, *Chilo suppressalis*; Ps, *Phyllotreta striolata*; Ag, *Anopheles gambiae*; Db, *Drosophila busckii*; Ad, *Anopheles darlingi*; Dm, *Drosophila melanogaster*; Si, *Solenopsis invicta*; Lm, *Locusta migratoria*; Dc, *Diaphorina citri*; Ago, *Aphis gossypii*.

**FIGURE 6:**
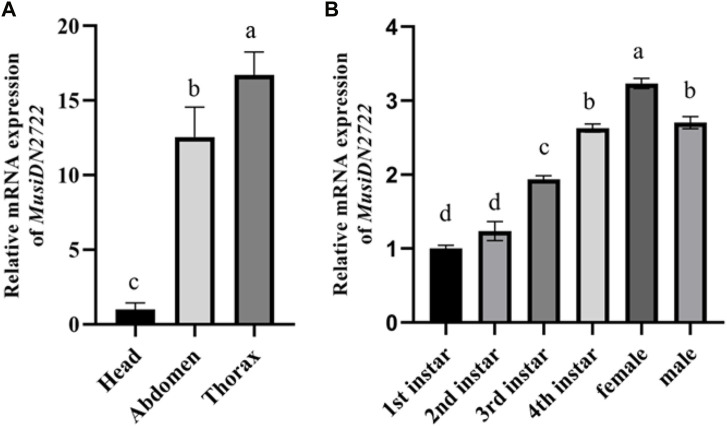
Relative levels of expression of *MusiDN2722* **(A)** in different adult tissues and **(B)** at different developmental stages of *M. usitatus*. Data are represented in the form mean ± SEM. Different letters indicate a significant difference at *p* < 0.05.

**FIGURE 7:**
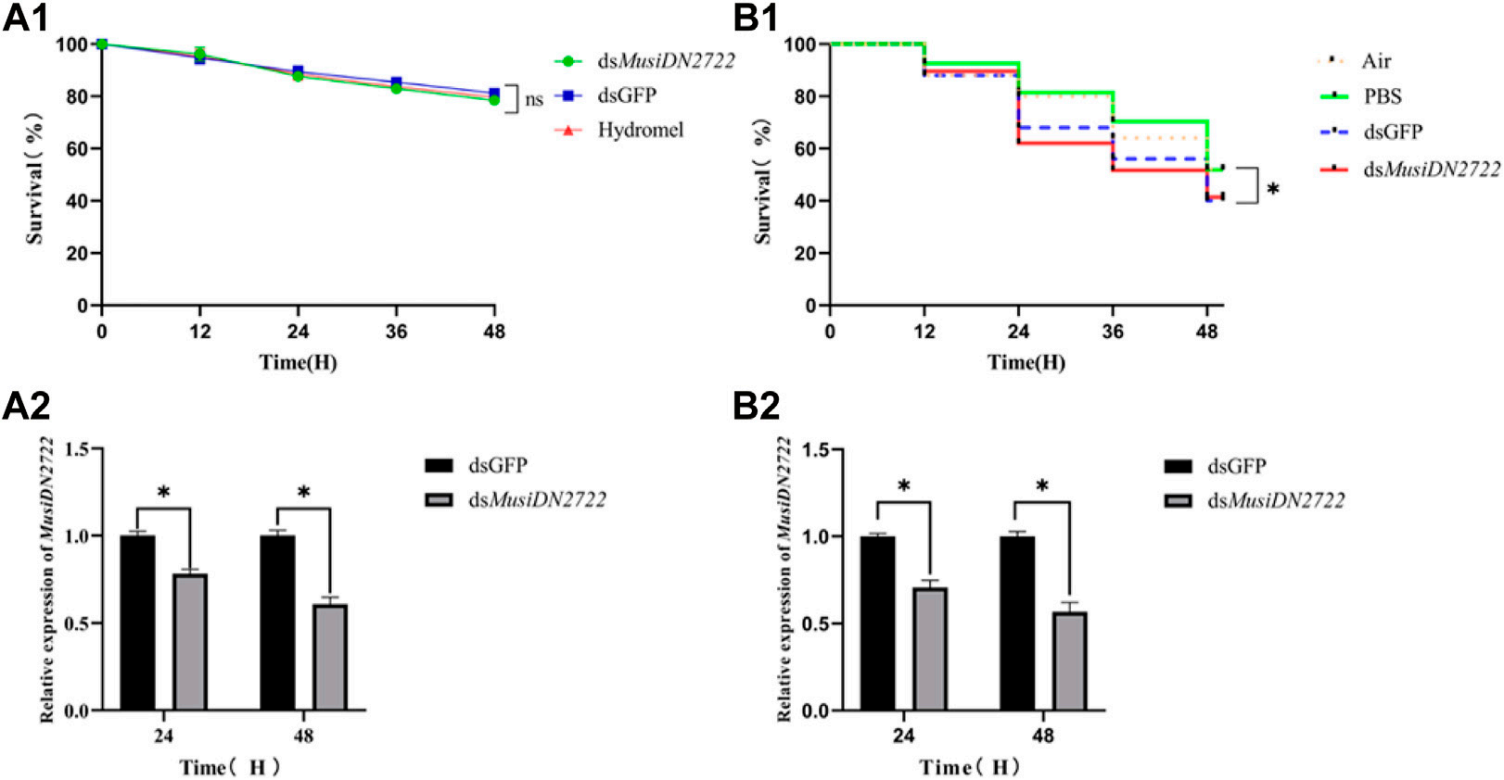
Survival rates after RNAi by different methods and gene expression of *MusiDN2722* at different times. **(A)** Feeding method; **(B)** injection method. Data are represented in the form mean ± SEM. *significant difference (*p* < 0.05).

RT-qPCR analysis of *M. usitatus* at different developmental stages indicated the expression of *MusiDN2722* in different instars. Expression was significantly higher during instars from the third larval stage to the adult stages than during the 1st and 2nd instar stages, and peaked during the 4th instar and adult stages. Expression was 1.67–3.23-fold higher at the 4th instar and female adult stages than at the other stages (Figures 5, 6A, 7). Expression in the head was used as a baseline (relative gene expression = 1) for comparison of gene expression in different tissues. The results of a relative gene expression test using RT-qPCR revealed that *MusiDN2722* was predominantly expressed in the thorax and abdomen, with levels 16.73- and 12.57-fold higher than the level in the head (Figure 6B).

### 3.4 Effects of membrane feeding and microinjection of ds*MusiDN2722* on ds*MusiDN2722* gene expression in *M. usitatus*

After 24 h, insects administered ds*MusiDN2722*, dsGFP, and 10% hydromel exhibited survival rates of 87.70%, 89.49%, and 88.54%, respectively. After 48 h, the survival rates were 78.53%, 81.19%, and 79.73%, respectively (Figure 7A1). At 24 and 48 h after microinjection for RNA interference, the survival rate of insects administered air (no injection solution), PBS, dsGFP, and ds*MusiDN2722* was assessed. As shown in Figure 6B, after 24 h, the survival rates of these insects were 80.00%, 81.48%, 68.00%, and 62.07%, respectively; after 48 h, the survival rates were 52.00%, 51.85%, 40.00%, and 41.38%, respectively. Analysis using SPSS revealed no significant difference between insects administered air and PBS or between those administered dsGFP and ds*MusiDN2722*, but the survival rate of the latter was considerably lower than that of the former (Figure 7B1).

The control group was fed and injected with dsGFP. RT-qPCR analysis showed that *MusiDN2722* expression in the membrane-fed insects, compared with that occurring in the control group fed dsGFP, was 21.76% and 39.21% after 24 and 48 h, respectively (Figure 7A2). This finding suggests that interference via the membrane-feeding method was effective to some degree. ds*MusiDN2722* was microinjected into the cavity of *M. usitatus*. At 24 and 48 h after the injection, gene expression was 29.35% and 43.20%, respectively, of that occurring in the control group, and the difference between the groups was significant (*p* < 0.05) (Figure 7B2).

### 3.5 *MusiDN2722* modulates acetamiprid resistance in *M. usitatus*

**FIGURE 8:**
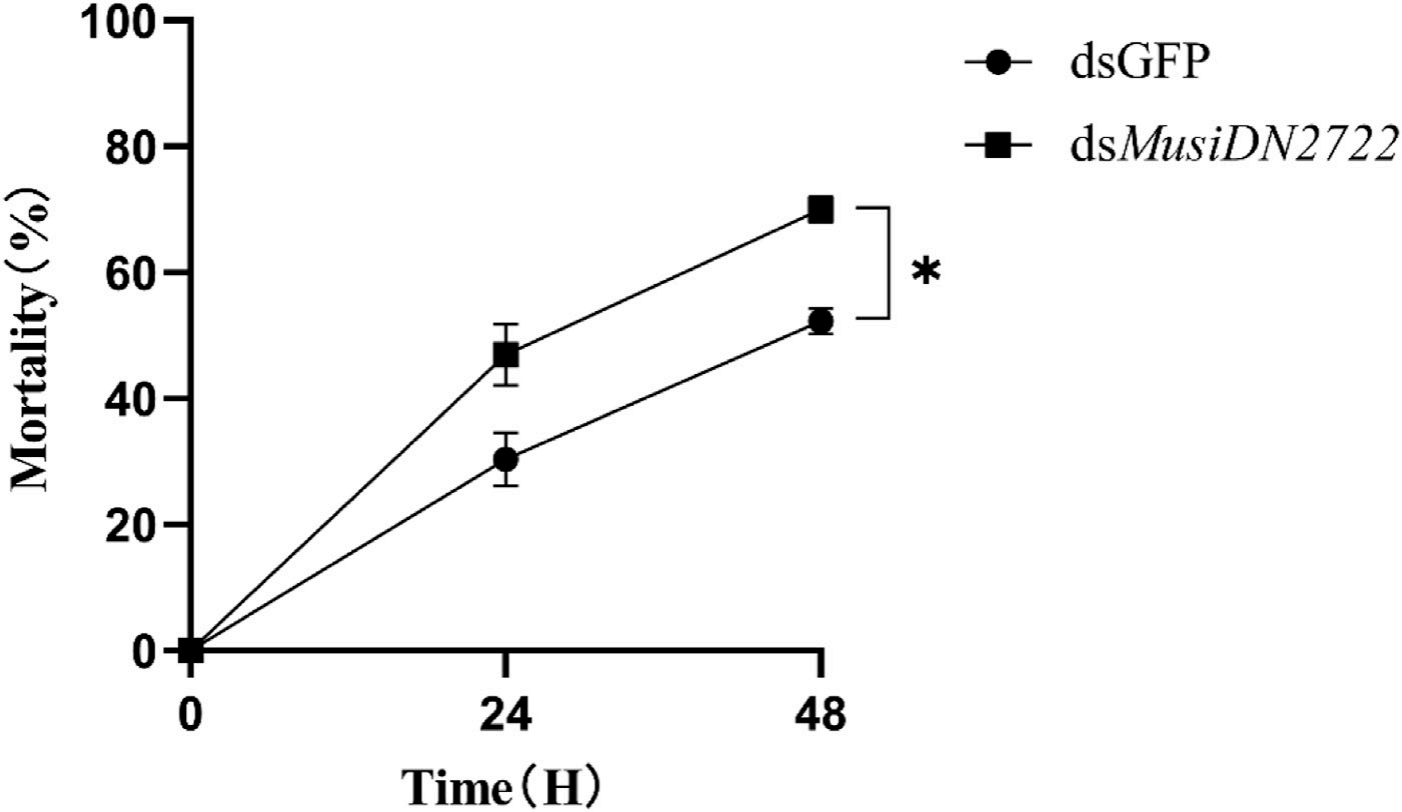
Mortality following dsRNA interference, 24 h and 48 h after treatment with acetamiprid LC_50_. Data are represented in the form mean ± SEM. *significant difference (*p* < 0.05).

The knockdown of *MusiDN2722* substantially increased the mortality of adults of the ACR strain relative to that of control adults upon exposure to 1878.99 mg/L acetamiprid. Toxicity bioassay showed that membrane-fed *M. usitatus* individuals were more susceptible to acetamiprid than the controls at 24 and 48 h (Figure 8). As shown in the figure, the mortality rate of the dsGFP group at 24 and 48 h was 30.38% and 52.31%, respectively, and that of the ds*MusiDN2722* group at 24 and 48 h was 46.98% and 68.29%, respectively. Compared with the dsGFP group, mortality increased by 23.4%.

### 3.6 Interacting protein prediction

**FIGURE 9:**
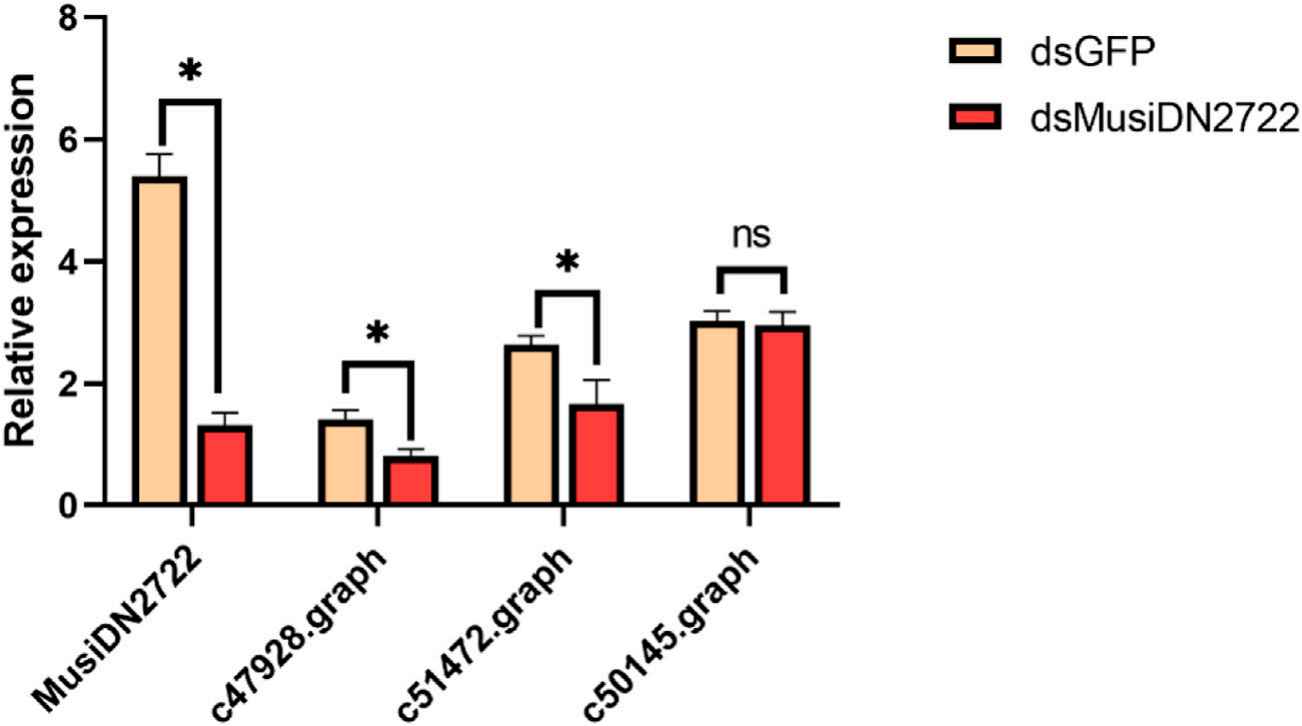
Expression of related genes after silencing of target genes.

Based on the results of prediction analysis using the STRING database, the corresponding genes were selected from the transcriptome for expression analysis. After silencing of *MusiDN2722*, there was a significant decrease in gene expression. CYP18A1, CYP12A4, and CYP4G15 correspond to transcripts C47928.graph, C51472.graph, and C50145.graph, respectively. According to the qPCR results, expression of C47928.graph and C51472.graph significantly decreased after silencing of *MusiDN2722*. C50145.graph exhibited no difference in expression before and after treatment (Figure 9).

## 4 Discussion

Owing to the widespread use of neonicotinoid insecticides for the control of *M. usitatus* and growing concerns regarding decreasing sensitivity, it is necessary to monitor resistance to these chemicals and elucidate the underlying mechanisms of resistance. *Megalurothrips usitatus* is a major threat to cowpea crops cultivated in Hainan and Guangdong provinces, China, and basic information on the mechanisms and pathways involved in insecticide resistance in *M. usitatus* is lacking. In this study, we observed increased susceptibility of *M. usitatus* to acetamiprid when expression of ds*MusiDN2722* was inhibited through RNA interference, indicating the possible involvement of *MusiDN2722* in the development of resistance and suggesting a possible target gene for genetic control of *M. usitatus*.

Monooxygenase-mediated detoxification is a common mechanism by which insects become resistant to insecticides (Scott, 1999). Studies have shown that the P450 genes related to insecticide resistance are mainly concentrated in the CYP3 (including CYP6 and CYP9) and CYP4 families (Feyereisen, 2012). In this study, the *MusiDN2722* sequence cloned from *M. usitatus* was used to construct a phylogenetic tree and was found to belong to the CYP6 clade, the family members of which have also been shown to play important roles in the detoxification and metabolism of toxic substances (Pan et al., 2018; Han et al., 2022). Through comparison with CYP genes of the same family in other insects, it was found that *MusiDN2722* shared the characteristic CYP450 sequence with other model insects, containing highly conserved hydrogen-bonding regions, including Helix-C (WxxxR), Helix-I (AGxxT), Helix-K (ExxR), Meander (FxxGxRxxxG), and the heme-binding domain (PxxFxPxxF) (Feyereisen, 2005).

The patterns of expression of detoxifying enzyme-coding genes at various stages of growth and development and in various tissues can, to a certain extent, reveal the functions of genes (Chung et al., 2009). These distinct expression patterns indicate that the proteins are involved in pesticide resistance and breakdown of secondary plant compounds (Ohkawa et al., 1999). Similarly to the findings of previous research (Xu et al., 2018; Hou et al., 2021), our findings revealed that *MusiDN2722* is expressed in both larvae and adults. For instance, it has been shown that mature worms (*Nilaparvata lugens*) express the P450 gene *CYP6ER1* (Mao et al., 2022). P450 activity differs between adult males and females. Adult females of *Culex pipiens quinquefasciatus* express the P450 gene at a higher level than adult males (Wang et al., 2014). This outcome was also validated in our research. Similarly, in *Chilo suppressalis*, the greatest level of expression of the P450 gene was observed in female adults (Bai et al., 2018). With age, thrips are increasingly exposed to damaging compounds in the external environment. To adapt to the environment, adults, particularly females, should be expected to express *MusiDN2722* at higher levels to detoxify and metabolize exogenous chemicals for survival (FENG, 2020). This is a form of adaptive evolution in insecticide resistance that occurs in mosquitoes. Additionally, the quantity and quality of P450 gene expression vary across insect tissues. If the P450 gene is overexpressed in the thorax and abdomen of resistant adults, it may be implicated in insect midgut detoxification (Zhao et al., 2021). The insect thorax may feature P450-related functional sites and binding sites (Yang et al., 2016). For instance, the P450 gene is essential for ecdysteroid production in the prothoracic gland of the silkworm *Bombyx mori* and the fruit fly *Drosophila melanogaster* (Ryusuke et al., 2004). These previous findings may explain why expression of *MusiDN2722* is substantially higher in the chest and abdomen than in the head. In contrast, other studies have shown that in insects such as *B. tabaci* (Liu et al., 2020) and *Lygus pratensis* (Ma et al., 2022), P450 expression is substantially higher in the head than in the chest and abdomen. These findings imply that the expression profiles of these genes are insect-specific.

Genes of the insect CYP6 subfamily play crucial roles in plant–insect interactions, particularly in the case of polyphagous insect pests (Nelson, 1998). Recently, putative functions of *CYP6AB14* and *CYP6AB60* in detoxifying harmful plant compounds in *Spodoptera litura* have been revealed (Pottier et al., 2012; Wang et al., 2015). The resistance of *B. tabaci* to neonicotinoid insecticides involves upregulation of *CYP6CM1* (Jones et al., 2011). In our study, silencing of *MusiDN2722* led to increased sensitivity of *M. usitatus* to acetamiprid, suggesting that *MusiDN2722* plays an important role in metabolizing neonicotinoid pesticides, thus affecting the toxicity tolerance of *M. usitatus*. Seventy-four CYP genes have been found in the potato beetle *Leptinotarsa decemlineata*, and six CYP6 family genes (*CYP6BH2*, *CYP6BJ1*, *CYP6BQ17*, *CYP6EG1*, *CYP6EH1*, and *CYP6EJ1*) are involved in the detoxification process of cyhalothrin (Wan et al., 2013). In our study, we found that *MusiDN2722* knockdown resulted in downregulation of *CYP18A1* and *CYP12A4*. This finding indicates that insect resistance may involve more than one detoxification enzyme. The involvement of more than one gene in insecticide resistance has also been reported in insects such as *Musca domestica* L. (Liu and Scott, 1996), *D. melanogaster* (Meigen) (Pedra et al., 2004), *Helicoverpa armigera* (Yang et al., 2006), and *Plutella xylostella* (Bautista et al., 2007).

Heritable RNAi through dsRNA expression is not possible in most insect species; therefore, loss-of-function experiments are mainly performed by introducing dsRNA from outside the insect body (Yu et al., 2013). In this study, *MusiDN2722* of *M. usitatus* was silenced using two methods: membrane feeding and microinjection. The results showed that interference in the gene of interest was successfully achieved *via* both methods. However, the survival rate of insects was significantly higher under the membrane-feeding method than under the microneedle injection method. This result is consistent with a previous finding (Prentice et al., 2017) and provides a technical means for the subsequent study of gene function in large thrips and small insects. These results also suggest that we need to consider various factors in future application processes to improve the effect of RNA interference. In fact, there are many other ways to deliver dsRNA, such the delivery of nucleic acid drugs (DNA or RNA) to insects via the targeted delivery and controlled release functions of nanocarriers (Nadeau, 2017; Zhang et al., 2021), but these methods may be challenging in the case of small insects such as thrips.

In the wild, insects usually detoxify toxins from plants by overexpressing detoxification enzymes. Although we have demonstrated using bioassays that P450-mediated detoxification certainly plays a major role in neonicotinoid resistance in *M. usitatus*, this does not rule out the possibility that other P450 monooxygenases and target-site resistance to imidacloprid might also play a role. However, several questions remain unanswered. It is still unknown how many different P450s contribute to resistance in a certain strain and how many significant amino acid changes occur in P450s, warranting further research.

## 5 Conclusion

In summary, *MusiDN2722* was identified as a P450-encoding gene in *M. usitatus*. RT-qPCR analysis revealed high *MusiDN2722* expression in females and in the thorax of *M. usitatus*. Both membrane-feeding and microinjection strategies successfully knocked down *MusiDN2722* and enhanced the sensitivity of common thrips to acetamiprid. Our findings lay a foundation for the determination of the long-term susceptibility of *M. usitatus* to neonicotinoid pesticides and for preservation of the field efficacy of this class of insecticides.

## Data Availability

The datasets presented in this study can be found in online repositories. The names of the repository/repositories and accession number(s) can be found below: NCBI, BankIt2661356 Seq1 OQ200384.
