# Supplementary figures and images for: RNA interference in cytochrome P450 monooxygenase (CYP) gene results in reduced insecticide resistance in Megalurothrips usitatus Bagnall
Source: Front Physiol. 2023 Mar 27;14:1130389. doi: 10.3389/fphys.2023.1130389 (PMC10083390; doi:10.3389/fphys.2023.1130389)

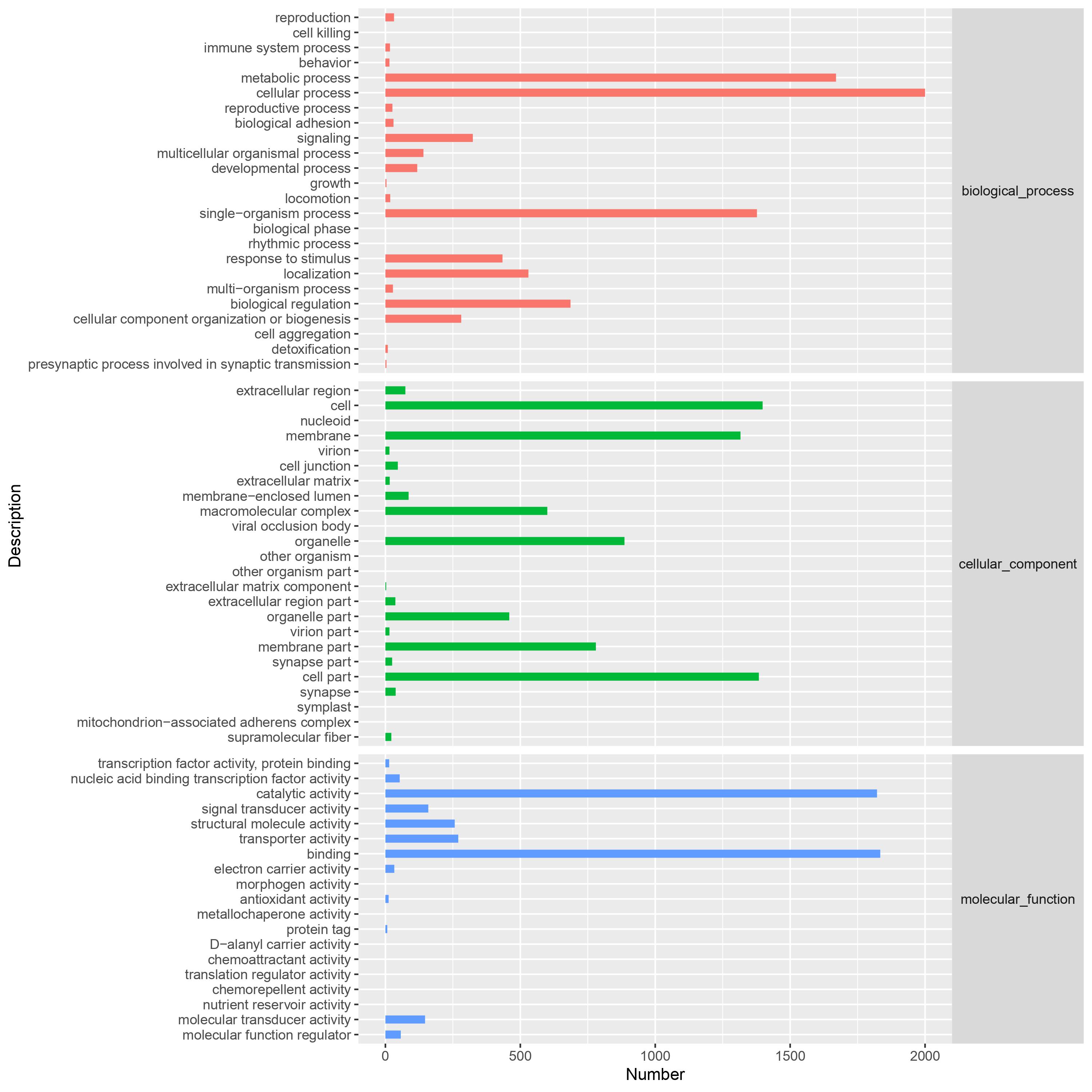

Supplement: Supplementary file 1 [file Image3.JPEG]

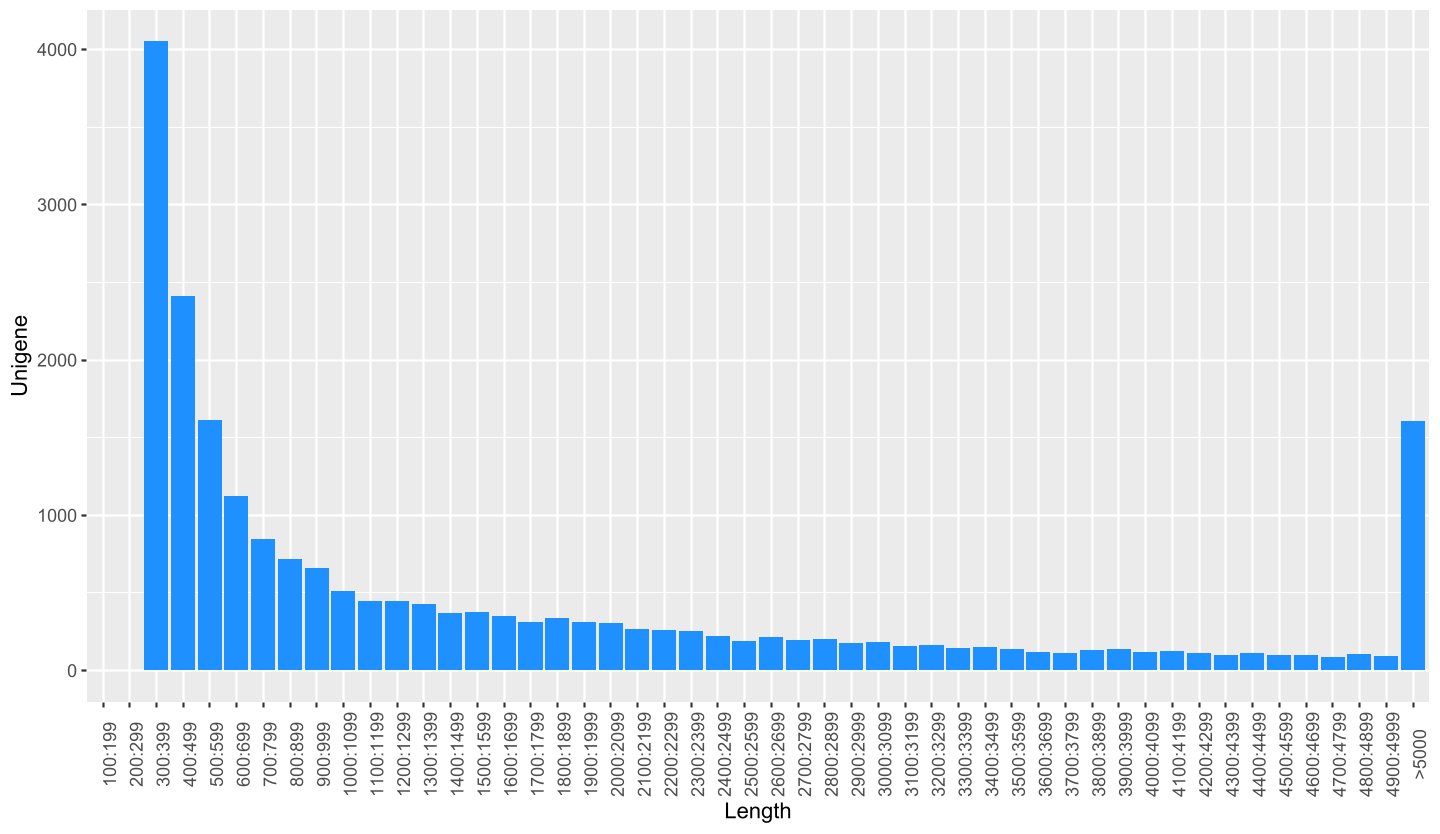

Supplement: Supplementary file 3 [file Image1.JPEG]

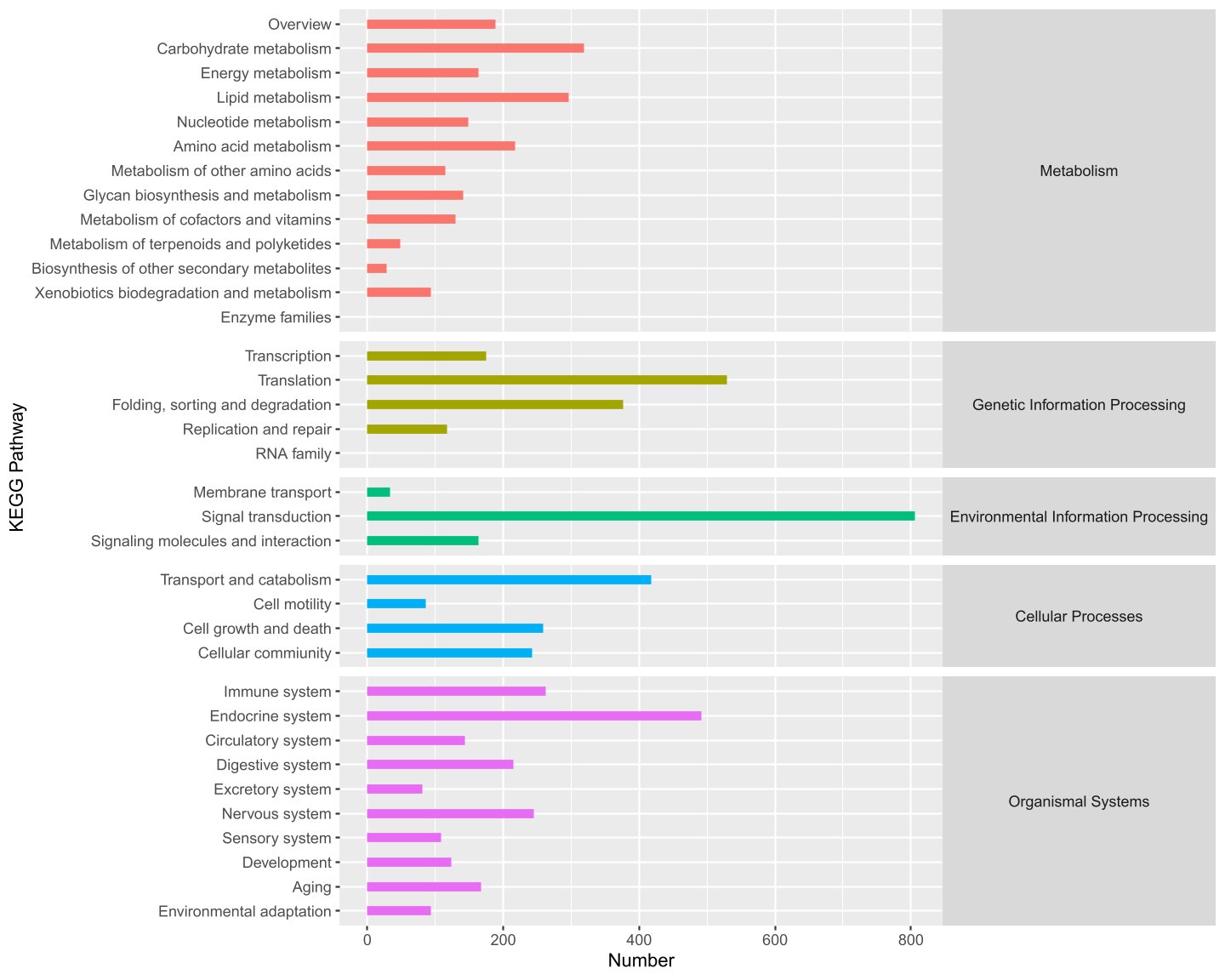

Supplement: Supplementary file 4 [file Image4.JPEG]

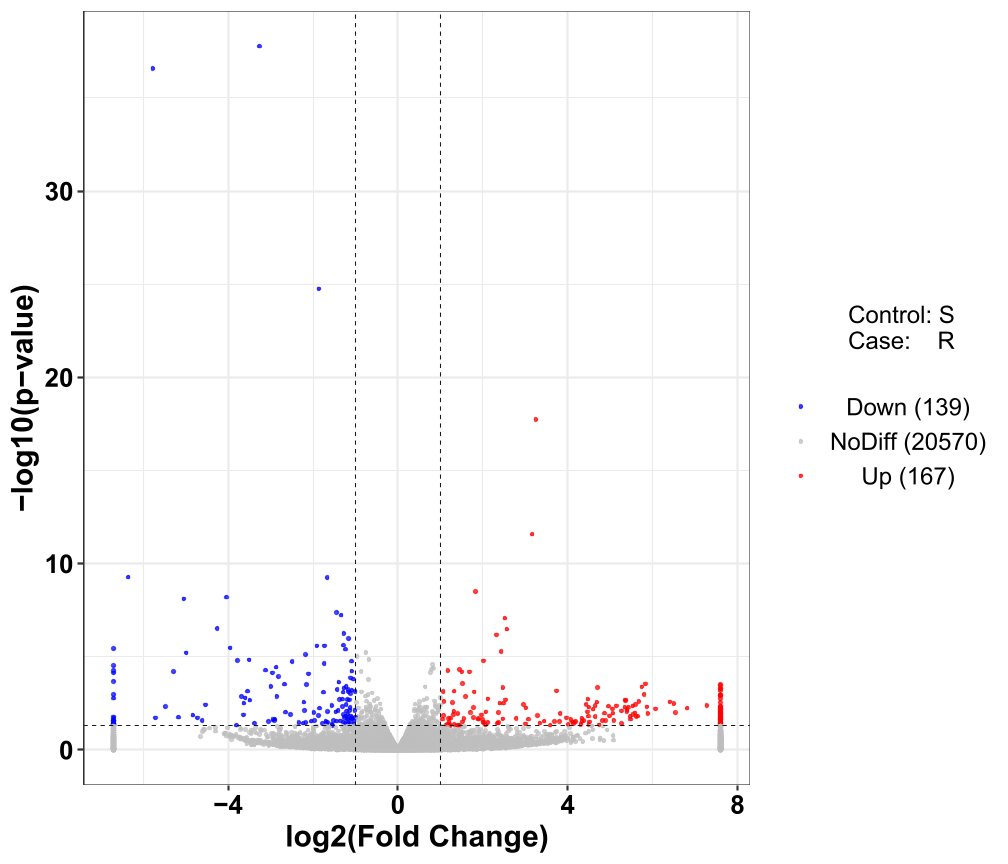

Supplement: Supplementary file 5 [file Image2.JPEG]
