# Supplementary material for: RNA interference in cytochrome P450 monooxygenase (CYP) gene results in reduced insecticide resistance in Megalurothrips usitatus Bagnall
Source: Front Physiol. 2023 Mar 27;14:1130389. doi: 10.3389/fphys.2023.1130389 (PMC10083390; doi:10.3389/fphys.2023.1130389)
Supplement: Supplementary file 2 [file Table1.DOCX]

Table 1. Primer sequences used in this study

| Primers | Sequence forward | Sequence reverse | Product |
| --- | --- | --- | --- |
| qMusiDN2722 | CCCTTCTTCGGCAACTTC | GCTTGCACCATTGGTAGAT | qPCR |
| CMusiDN2722 | CCCTCGTCTACCTGTTCTTCT | CTCGACGCGCAGCTTAAA | Clone |
| dsMusiDN2722 | TAATACGACTCACTATAGGGTGCTGAGGGACTTCACCTTC | TAATACGACTCACTATAGGGTTAATGGACACGCTCTGTCG | RNA interference |
| GAPDH | ACTGTTGATGGTCCCTCTGG | AGCGGCTTCCTTAACCTTCT | qPCR |
| dsGFP | TAATACGACTCACTATAGGGGGGCACAACAGACAATC | TAATACGACTCACTATAGGGGGCTTCCATCCGAGTA | RNA interference |
| M13 | CAGGGTTTTCCCAGTCACG | GAGCGGATAACAATTTCACAC |  |
| C47928 | CTCCAGGTACTTCATCTCCA | GGTCGACACCATCATGTTC | qPCR |
| C51472 | CATCGCATCCAGTGAAGAG | TCCTAAAGTGACCCAGATACA | qPCR |
| C50145 | CTCCAGGTACTTCATCTCCA | GGTCGACACCATCATGTTC | qPCR |
